# Quantitative Analysis of Diffusion-Restricted Lesions in a Differential Diagnosis of Status Epilepticus and Acute Ischemic Stroke

**DOI:** 10.3389/fneur.2022.926381

**Published:** 2022-07-07

**Authors:** Lukas Machegger, Pilar Bosque Varela, Giorgi Kuchukhidze, Jürgen Steinbacher, Andreas Öllerer, Tanja Prüwasser, Georg Zimmermann, Slaven Pikija, Johannes Pfaff, Eugen Trinka, Mark Mc Coy

**Affiliations:** ^1^Department of Neuroradiology, Christian Doppler University Hospital, Paracelsus Medical University, Salzburg, Austria; ^2^Department of Neurology, Christian Doppler University Hospital, Member of the European Reference Network EpiCARE, Paracelsus Medical University of Salzburg, Salzburg, Austria; ^3^Centre for Cognitive Neuroscience Salzburg, Neuroscience Institute, Christian Doppler University Hospital, Salzburg, Austria; ^4^Department of Mathematics, Paris-Lodron University, Salzburg, Austria; ^5^IDA Lab Salzburg, Team Biostatistics and Big Medical Data, Paracelsus Medical University, Salzburg, Austria; ^6^Research and Innovation Management, Paracelsus Medical University, Salzburg, Austria; ^7^Karl Landsteiner Institute for Neurorehabilitation and Space Neurology, Salzburg, Austria

**Keywords:** status epileptics, diffusion restriction, MRI, acute ischemic stroke, quantification

## Abstract

**Background and Purpose:**

Distinction between acute ischemic stroke (AIS) and status epilepticus (SE) on MRI can be challenging as restricted diffusion may occur in both conditions. In this study, we aimed to test a tool, which could help in differentiating AIS from SE when restricted diffusion was present on MRI.

**Materials and Methods:**

In diffusion weighted imaging (DWI) with a b-value of 1,000 and apparent diffusion coefficient (ADC) maps, we compared the ratios of intensities of gray values of diffusion-restricted lesions to the healthy mirror side in patients with AIS and SE. Patients were recruited prospectively between February 2019 and October 2021. All patients underwent MRI and EEG within the first 48 h of symptom onset.

**Results:**

We identified 26 patients with SE and 164 patients with AIS. All patients had diffusion-restricted lesions with a hyperintensity in DWI and ADC signal decrease. Diffusion restriction was significantly more intense in patients with AIS as compared to patients with SE. The median ratios of intensities of gray values of diffusion-restricted lesions to the healthy mirror side for DWI were 1.42 (interquartile range [IQR] 1.32–1.47) in SE and 1.67 (IQR 1.49–1.90) in AIS (*p* < 0.001). ADC decrease was more significant in AIS as compared to SE with median ratios of 0.80 (IQR 0.72–0.89) vs. 0.61 (IQR 0.50–0.71), respectively (*p* < 0.001). A cutoff value for ratios of DWI signal was 1.495 with a sensitivity of 75% and a specificity of 85%. Values lower than 1.495 were more likely to be associated with SE and higher values were with AIS. A cutoff value for ADC ratios was 0.735 with a sensitivity of 73% and a specificity of 84%. Values lower than 0.735 were more likely to be associated with AIS and higher values were with SE.

**Conclusion:**

Diffusion restriction and ADC decrease were significantly more intense in patients with AIS as compared to SE. Therefore, quantitative analysis of diffusion restriction may be a helpful tool for differentiating between AIS and SE when restricted diffusion is present on MRI.

## Introduction

Status epilepticus (SE) is a maximum expression of seizure, which can be associated with or caused by various disorders. SE is defined as “a condition resulting either from the failure of the mechanisms responsible for seizure termination or from the initiation of mechanisms, which lead to abnormally prolonged seizures (after time point t1). It is a condition, which can have long-term consequences (after time point t2), including neuronal death, neuronal injury, and alteration of neuronal networks, depending on the type and duration of seizures” (1).

This definition of SE underlines the pathophysiologic mechanisms that sustain the development of SE and its perpetuation, increasing the risk of neuronal injury with time (1). In SE, both vasogenic and cytotoxic brain edema may occur, which makes it difficult to differentiate SE from an acute ischemic stroke (AIS) on MRI in the absence of large vessel occlusion and based only on diffusion-weighted images/apparent diffusion coefficient (DWI/ADC) values. The timing of the appearance of two types of edemas and their roles in developing long-term consequences in SE is not well-established. There are several suggestions, which may help in discriminating SE from AIS if diffusion restriction occurs on MRI: (1) the alterations related to SE do not respect brain arterial vascular territories (2). (2) Diffusion restriction in SE may occur simultaneously with an increased signal on T2-weighted imaging (such as fluid-attenuated inversion recovery [FLAIR]). In AIS, diffusion restriction usually precedes T2-weighted alterations. (3) MRI sequences demonstrating cerebral blood perfusion play a crucial role in differentiating ongoing seizure activity (associated with increased perfusion) from AIS (hypoperfusion) (3). However, luxury perfusion – an excessive blood flow in an infarcted brain area due to dysfunctional autoregulation and permeability of the blood–brain barrier – may occur (4, 5). It commonly occurs in subacute stroke (>72) in treated and untreated cases, but it has been reported also in the first 24 h (5). SE-related hyperperfusion, in contrast to luxury perfusion, is not limited to a single vessel territory and occurs frequently before diffusion restriction on DWI (4). The signal intensities on DWI and ADC are not as prominent in SE as in AIS. This topic, however, has not been yet systematically studied.

In this study, we aimed to propose an additional tool for differentiating SE from AIS on MRI by quantifying diffusion restriction and suggesting cutoff values of DWI signal intensities in patients with SE as compared to those with AIS.

## Methods

This is a case-control monocentric study conducted on adult patients (older than 18 years) with SE (index group) and those with AIS (control group). Patients were recruited prospectively and examined at the Department of Neurology and the Department of Neuroradiology, Christian Doppler University Hospital, Paracelsus Medical University of Salzburg, Austria between 20 February 2019 and 13 October 2021. The data analysis was done retrospectively. All patients underwent EEG and cranial MRI within the first 48 h after clinical onset. In the index group, we included patients with an electro-clinical diagnosis of SE who had diffusion-restricted lesions on MRI. Seven patients with global cerebral hypoxia due to cardiac arrest were excluded. In the control group, patients with AIS were included if along with an MRI they underwent an EEG for ruling out SE, seizure patterns, and epileptiform discharges. The AIS group was defined based on the following criteria: (1) patients without large vessel occlusion; (2) patients without attempted reperfusion therapies; and (3) patients without known history or currently taking medications, which may cause diffusion restriction.

In the AIS group, a median time lapse between an MRI and an EEG was 5.6 h (interquartile range [IQR] 2.8–24.4). In the SE group, a median time lapse between an MRI and an EEG was 8.5 h (IQR 3.9–24.1).

All patients underwent an MRI at the Department of Neuroradiology, Christian Doppler University Hospital, Paracelsus Medical University of Salzburg, Austria on a 3T machine, Achieva dStream (Philips Medical Systems, Best, Netherlands).

The following parameters of DWI sequence were employed: spin echo-EPI diffusion imaging was used to acquire 28 slices with echo time (TE) 47 ms, repetition time (TR) 3,051 ms, field of view (FOV) 230 × 230 mm, and voxel 2.05 × 2.56 mm, with a slice thickness of 4 mm and a gap between the slices of 1 mm. The diffusion sequence was acquired with four b-values of 0, 333, 666, and 1,000 s/mm^2^. Diffusion gradients were applied in three directions. All patients in the study (those with AIS and SE) underwent the same DWI sequence.

We used 4 b-values for calculating the conventional ADC map, which is based on the logarithm of signal intensity of the diffusion image divided by the signal intensity of the image without diffusion gradient ADC = −1/b ln(S_DWI_/S_0_).

We compared intensities of gray values of diffusion-restricted lesions to the healthy mirror side in DWI slices with a b-value of 1,000 and in ADC maps. The size of the region of interest (ROI) was chosen to cover the maximum signal intensity of the lesion; an equal size was used for the mirror side. In the case of anatomic asymmetry of brain hemispheres, the reference ROI on the healthy side was chosen in the most corresponding anatomical region. A target lesion with the maximum signal intensity in DWI, b = 1,000, was selected for each patient. The resulting average gray value intensity of the selected area was compared between a lesion and a healthy side.

The quantification of the DWI-restricted lesions was done by drawing manually circular ROIs using IntelliSpace Portal version 10.1.

We calculated and compared ratios of signal intensity values of DWI and ADC on the lesion side to the healthy mirror side for both groups of patients (SE and AIS). Furthermore, based on these ratios, we calculated a cutoff value for differentiating between AIS and SE in DWI with a b-value of 1,000 and in ADC maps.

In all patients, a standard 30-min EEG based on the 10–20 international system was performed.

### Statistics

All data were analyzed using SPSS 27.0.1.0 statistical software. Comparisons of SE and AIS groups with regard to DWI and ADC signal ratios were performed by means of the Mann-Whitney U test. Significance was set at *p* ≤ 0.05. Optimal cutoff values for DWI and ADC signal ratios were determined by the use of Youden's index. There were no missing data in the entire analysis.

## Results

### Study Population

We enrolled 338 patients with a diagnosis of SE at our institution. MRI was done in 199 of 338 (59%) patients. In 153 of 199 (77%) of these patients, MRI was performed within 48 h after the onset of SE. Diffusion restriction was observed on MRI in 26 of 146 (18 %) patients with an SE (16 women; median age 68 years, IQR 54.5–76.5).

During the same period, 503 patients with suspected AIS showed diffusion-restricted lesions on MRI and underwent an EEG for ruling out an SE, seizure pattern, or an epileptiform activity. The patients were excluded from the study if (1) they underwent an MRI on a different machine rather than 3T Achieva dStream, Philips Medical Systems, Best, the Netherlands (22 patients), (2) an ictal or interictal epileptiform activity was seen on EEG (11 patients), and (3) there was a clinical-radiological discordance with AIS suspected on initial MRI and a different diagnosis rather than AIS upon discharge from the hospital (85 patients). Thus, 385 patients with AIS were selected. However, only 164 of 385 (43%) patients had an MRI within the first 48 h after symptom onset (65 women; the median age of 75 years, IQR 66–80).

Demographic data of patients with SE and AIS are shown in Tables 1, 2. For AIS, the Trial of Org 10,172 in Acute Stroke Treatment (TOAST) classification was used (6). Median National Institute of Health Stroke Scale (NIHSS) at admission was 4.0 (IQR 2.0–8.0) and 2.0 (IQR 0.0–5.0) at discharge from the hospital. Median modified Rankin Scale (mRS) at admission was 3.0 (IQR 2.0–4.0), at discharge −2.0 (IQR 0.0–5.0).

**Table 1:** Demographic, clinical, and MRI characteristics of patients with SE.

**Status epilepticus**	**Total *N* = 26**
Median age (IQR)	68 years (54.5–76.5)
Gender (Female/Male)	16 (61%) /10 (39%)
PMA location	Number of lesions (%)
Frontal cortex	6 (23)
Parietal cortex	6 (23)
Temporal cortex	4 (15)
Occipital cortex	1 (4)
Amygdala/Hippocampus	5 (19)
Pulvinar of thalamus	2 (8)
Others	2 (8)
SE type	Number of patients (%)
NCSE	17 (66)
CSE	5 (19)
CSE-NCSE	4 (15)
SE etiology	Number of patients (%)
Remote cerebrovascular disease	6 (23)
Head trauma	5 (19)
Cryptogenic	3 (11.5)
Intracranial tumor	3 (11.5)
Metabolic disturbance	2 (8)
Autoimmune	2 (8)
Others	5 (19)
EMSE, median (IQR)	58 (26.25–72.5)
SE duration, median (IQR)	3.0 (1.6–24.0)
Time to MRI, median (IQR)	10.0 (2.0–25.5)

*PMA, peri-ictal MRI abnormalities; CSE, convulsive SE; NCSE, non-convulsive SE; EMSE, epidemiology-based mortality score; SD, standard deviation; IQR, interquartile range*.

**Table 2:** Demographic and clinical variables of stroke patients.

**Stroke**	**Total *N* = 164**
Median age (IQR)	75 years (66–80)
Gender (Female / Male)	65 (40%) / 99 (60%)
Stroke TOAST classification	Number of patients (%)
Stroke of undetermined etiology	46 (28)
Cardioembolism	44 (27)
Large-artery artherosclerosis	43 (26)
Small vessel occlusion	22 (13)
Stroke of other determined etiology	9 (6)
Stroke territory	Number of patients (%)
Middle cerebral artery (MCA)	118 (72)
Posterior cerebral artery (PCA)	21 (13)
Anterior cerebral artery (ACA)	8 (5)
Others (PICA, AICA, SCA, and BA)	17 (10)
NIHSS at admission/discharge	Number of patients (%)
<5	101 (62) /124 (76)
5–10	34 (21) / 18 (11)
11–20	22 (13) / 19 (12)
>20	7 (4) / 3 (2)
mRS at admission/discharge	Number of patients (%)
0	13 (8) / 31 (19)
1–3	71 (43) / 81 (50)
>3	80 (49) / 52 (32)
Median time to MRI (IQR)	12.8 (4.1–27.4)

*TOAST, Trial of Org 10172 in Acute Stroke Treatment; PICA, posterior inferior cerebellar artery; AICA, anterior inferior cerebellar artery; SCA, superior cerebellar artery; BA, basilar artery; NIHSS, National Institute of Health Stroke Scale; mRS, modified Rankin Scale; IQR, interquartile range*.

The median time from diagnosis to MRI for patients with AIS was 12.8 h (IQR 4.1–27.4). The most frequent etiologies for AIS were cryptogenic −46 (28%), cardioembolic −44 (27%), and atherothrombotic −43 (26%). For details of other etiologies, see Table 2.

Etiologies of SE were various; the most frequent was SE due to remote cerebrovascular disease (23%). The median time to MRI for the SE group was 10.0 h (IQR 2.0–25.5). The median duration of SE was 3.0 h (IQR 1.6–24.0). The median epidemiology-based mortality score in status epilepticus [EMSE (7)] was 58 (IQR 26.25–72.5).

### Diffusion Restriction in SE vs. AIS

**Figure 1:**
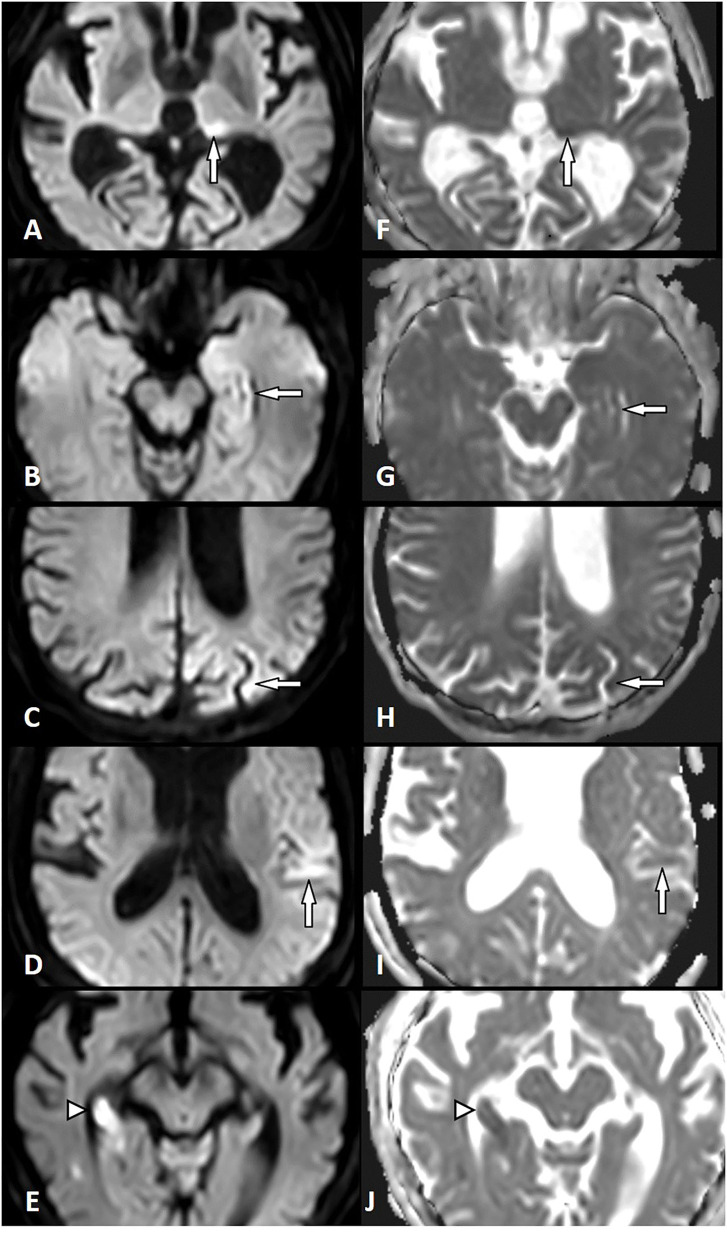
Diffusion restriction in status epilepticus (SE) and acute ischemic stroke (AIS). Left column: Diffusion-weighted imaging (DWI). Right column: Apparent diffusion coefficient (ADC). SE-related diffusion restriction in the left pulvinar **(A,F)**, in the left hippocampus **(B,G)**, the left parietal cortex **(C,H)**, and the left temporal cortex **(D,I)**. AIS-related diffusion restriction in the right hippocampus (right posterior cerebral artery territory) **(E,J)** (white arrow, SE lesion; white arrowhead, AIS lesion).

**Figure 2:**
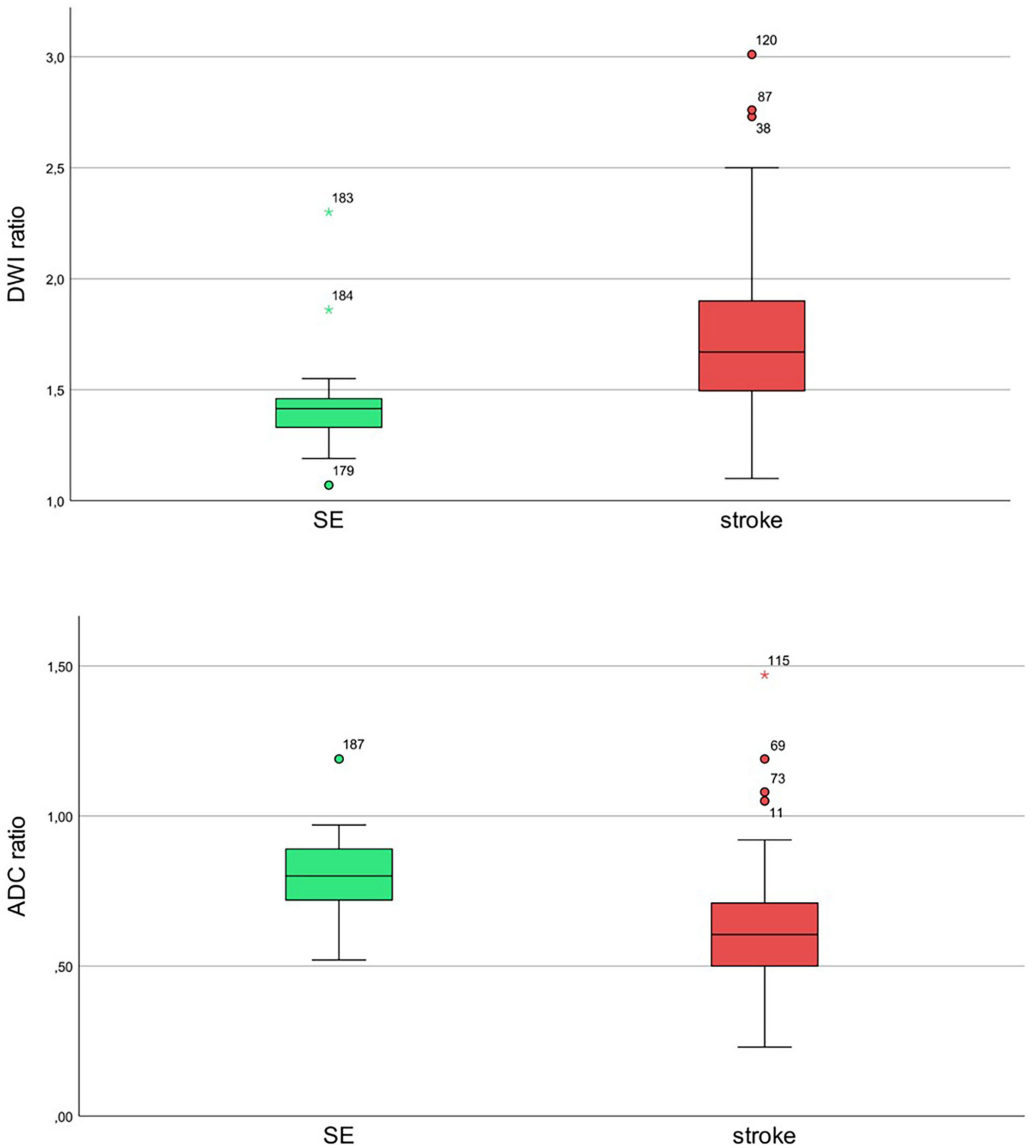
Ratios of diffusion-restricted lesions to mirror the healthy side for status epilepticus (SE; green) and stroke (red) with diffusion-weighted imaging (DWI) hyperintensity and apparent diffusion coefficient (ADC) signal decrease for SE and stroke patients. Upper image: diffusion b-value of 1,000. Lower image: ADC maps.

All patients with AIS (*n* = 164) and SE (*n* = 26) showed diffusion restriction with hyperintensity in DWI and an ADC signal decrease (Figure 1). However, diffusion restriction was significantly more intense in patients with AIS as compared to patients with SE (Figure 2) with median ratios of the signal intensity for DWI restriction of 1.42 (IQR 1.32–1.47) in SE and 1.67 (IQR 1.49–1.90) in AIS (*p* < 0.001). ADC signal decrease was more significant in AIS as compared to SE (Figure 2) with median values of ratios of signal intensity −0.80 (IQR 0.72–0.89) vs. 0.61 (IQR 0.50–0.71), respectively (*p* < 0.001).

We calculated cutoff values for the ratios of signal intensity of gray values for DWI and ADC in order to differentiate between these two groups.

**Figure 3:**
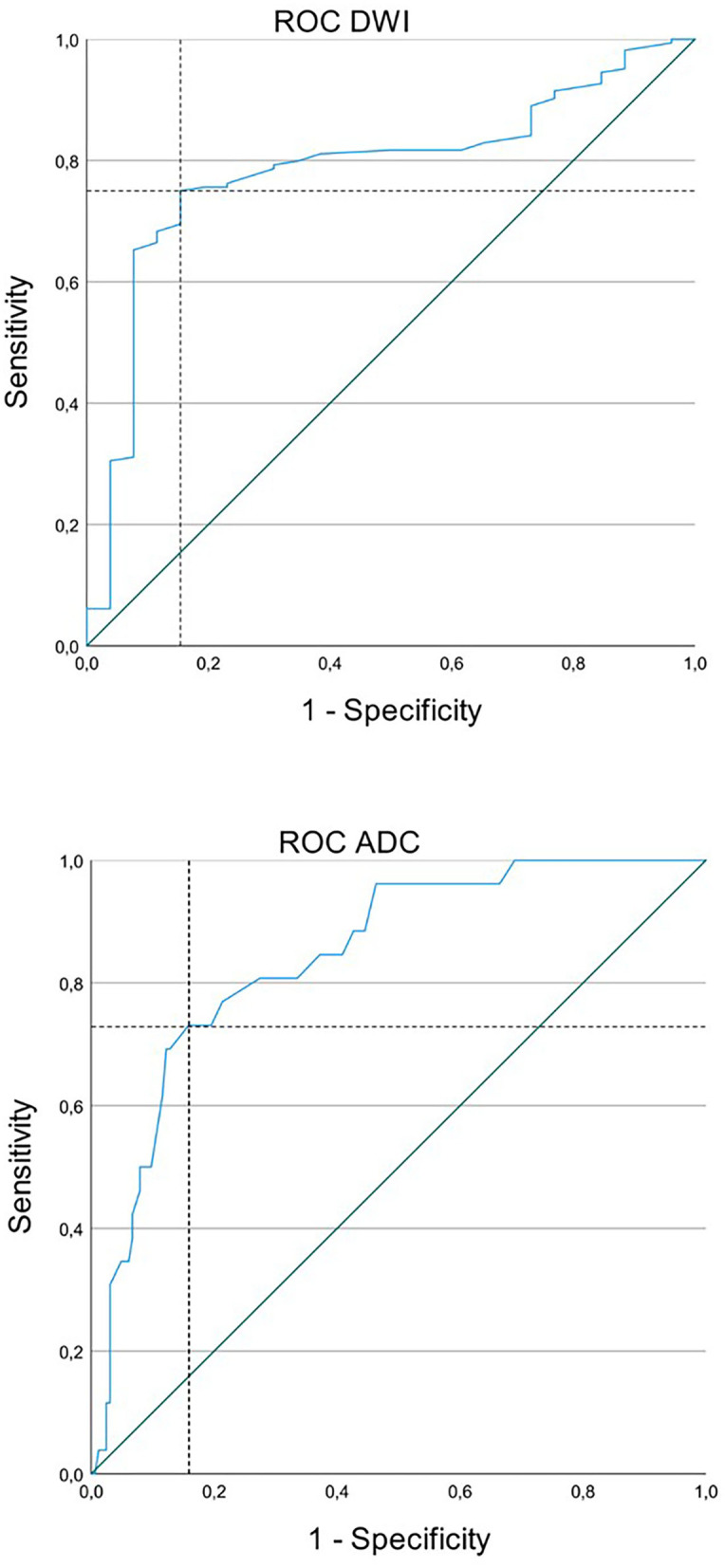
Receiver operating characteristic (ROC) curve for status epilepticus (SE) and acute ischemic stroke (AIS) with diffusion restriction and apparent diffusion coefficient (ADC) signal decrease. Upper image: The cutoff value for diffusion-weighted imaging (DWI) was 1.495 with a sensitivity of 75.0% and a specificity of 84.6%. Lower image: The cutoff value for ADC was 0.745 with a sensitivity of 73.1% and a specificity of 84.1%.

The cutoff value, the point on the curve with the smallest distance to the point of highest sensitivity and specificity (top left corner), is marked in Figure 3 with the dashed line.

**Figure 4:**
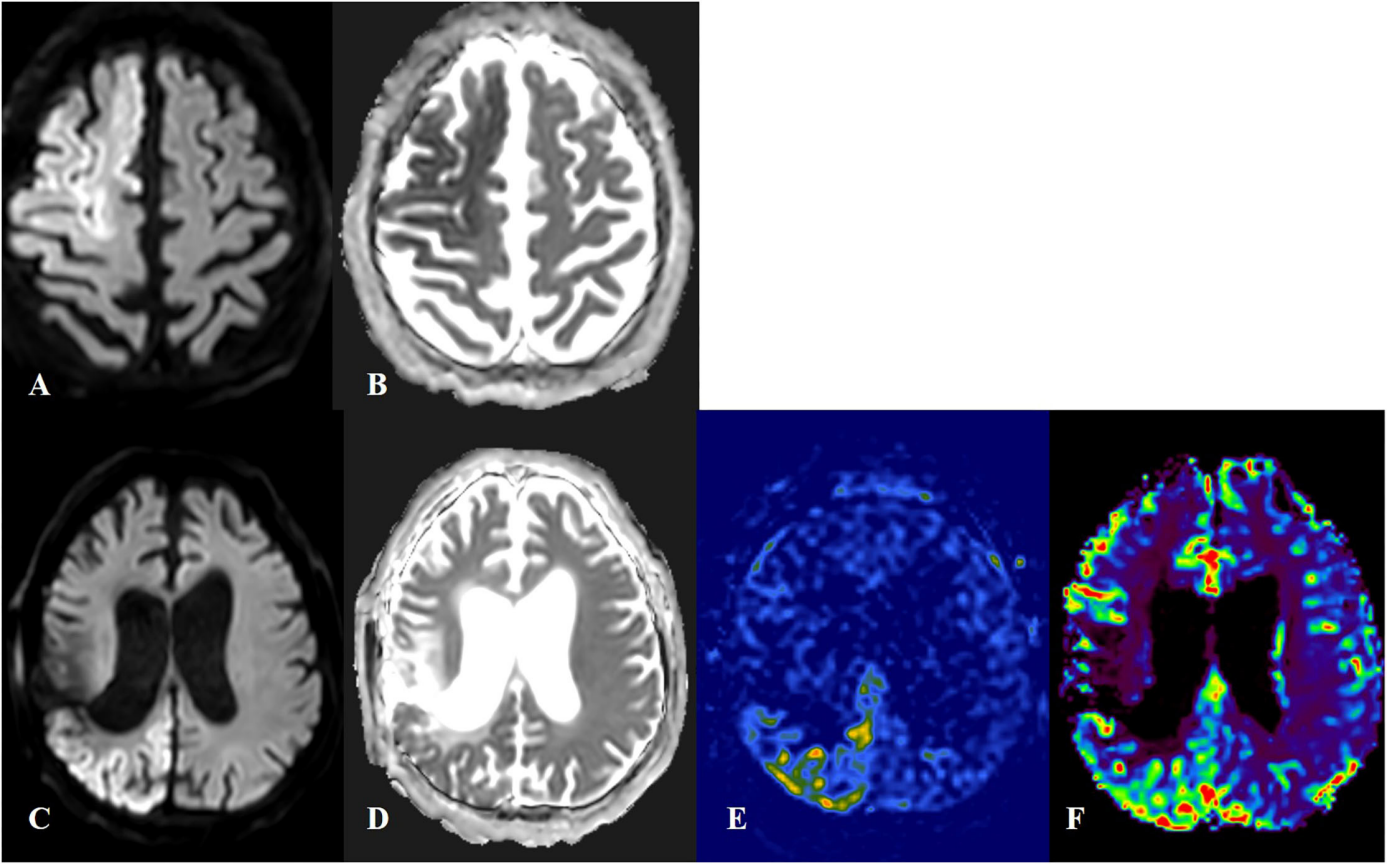
Cortical laminar necrosis in acute ischemic stroke (AIS) and status epilepticus (SE). First row: Patient with AIS in territories of right anterior and middle cerebral arteries, with an increased signal in diffusion-weighted imaging (DWI) **(A)** and decreased signal in apparent diffusion coefficient (ADC) **(B)**. Quantification of DWI and ADC signals was done for the lesion side as compared to the contralateral healthy mirror side. The ratio for DWI lesion/healthy side was 1.70 and the ratio for ADC lesion/healthy side was 0.58. Second row: Patient with SE. The DWI-restricted lesion in the right parieto-occipital region **(C,D)** was suggestive of peri-ictal MRI abnormalities. In arterial spin labeling **(E)** and MRI-perfusion with a contrast substance **(F)**, an ictal hyperperfusion in the right parieto-occipital region was observed. The ratio for DWI lesion/healthy side was 1.40 and the ratio for ADC lesion/healthy side was 0.84.

A cutoff value for ratios of DWI signal intensity was 1.495 with a sensitivity of 75% and a specificity of 85%. Values lower than 1.495 were more likely to be associated with SE and values higher than 1.495 were with AIS. A cutoff value for ADC signal intensity was 0.735 with a sensitivity of 73% and a specificity of 84%. Values lower than 0.735 were more likely to be associated with AIS and values higher than 0.735 were with SE (Figures 3, 4).

## Discussion

In this study, we compared quantified DWI and ADC signal intensities in patients with SE and AIS and proposed cutoff values, which may be useful for differentiating between these two entities on MRI. We could demonstrate that patients with AIS had significantly higher intensity of a DWI signal and lower ADC values as compared to those with a SE in the first 48 h after the onset of symptoms. Cutoff values for both DWI and ADC signals had relatively high sensitivity and specificity.

MRI is crucial in a diagnostic work-up of SE. Variable peri-ictal MRI alterations (8, 9) have been reported in patients with SE, in the ictal or in the early post-ictal period. Restricted diffusion (9, 10) and hyperintensities in T2-weighted and FLAIR sequences that could even appear simultaneously with diffusion restriction (11) are the most frequently encountered MRI alterations in SE. These changes represent a continuum of cytotoxic and vasogenic edema mostly depending upon the timing of MRI performance (8, 9). Ictal hyperperfusion in MR perfusion sequences (3) and leptomeningeal contrast enhancement may also be seen in patients with SE (9, 12). The majority of imaging studies show that there are particular brain areas, which are usually affected in patients with SE: the neocortex, the hippocampus, the pulvinar of the thalamus, the splenium of the corpus callosum, and the contralateral cerebellum (2, 13). MRI changes related to SE may be completely reversible (14). However, they can also persist and be followed by permanent alterations, such as cortical laminar necrosis, mesial temporal sclerosis (8, 14, 15), and focal brain atrophy (9, 11). Peri-ictal DWI abnormalities (especially in mesial temporal structures) are most frequently associated with permanent alterations, thus representing a predictive factor for an unfavorable outcome of SE. Acute DWI changes in SE may also serve as a source for localizing the seizure onset zone. The longer the seizure or SE, the greater the chances of developing diffusion restriction on MRI (3, 16).

Epileptic seizure and especially SE are the most common stroke mimics. They represent 2–30% of patients admitted to a stroke unit (17). Most adult patients with suspected stroke-mimics have either a remote symptomatic epileptic seizure or a non-convulsive SE (18). Non-convulsive SE is frequently difficult to differentiate from AIS on MRI as diffusion restriction may occur in both conditions. This problem becomes more complicated in patients with symptomatic SE due to a chronic post-ischemic lesion. Frequently, a new stroke is suspected if an area with a diffusion restriction is observed adjacent to an old post-stroke lesion. Here, some other MRI sequences may give clues for a correct diagnosis, especially if an EEG is non-conclusive. MR-perfusion studies usually demonstrate hyperperfusion of the brain tissue in the case of SE, as was shown in two patients with equivocal EEG and diffusion restriction in the vicinity of an old stroke lesion (19).

Cortical diffusion restriction, presented by a high signal in DWI and low ADC values in SE, may resemble cortical laminar necrosis due to ischemic stroke. Follow-up MRIs should be performed, as SE-associated cortical diffusion restriction is commonly reversible as opposed to the cortical laminar necrosis due to cerebral infarction (20).

Our study is a first attempt to quantify DWI and ADC signal intensities in patients with SE as compared to those with AIS. Quantification of DWI and ADC signals has been widely performed in patients with stroke mainly for determining the age of the infarction or for predicting clinical outcomes (21, 22). It has been observed that diffusion restriction occurring after a single seizure or SE is usually not as prominent as in acute stroke, however, quantification analysis of DWI and ADC signal has not been performed (23).

In our cohort, in both groups of patients (SE and AIS), diffusion restriction was observed with hyperintensity in DWI and low ADC values, reflecting cytotoxic edema. In SE, however, both vasogenic and cytotoxic brain edema may occur. As opposed to cytotoxic edema, vasogenic edema is related to an increase in extracellular water content and a breakdown of the blood-brain barrier. Cytotoxic edema occurs due to intracellular accumulation of liquid because of the failure of an ATP-dependent Na+/K+ membrane pump and the intracellular influx of Ca++ (24). This mechanism is similar to SE and AIS. Another possible pathomechanism of cytotoxic edema in epilepsy patients could be related to an increased release of glutamate and other excitatory neurotransmitters during ongoing seizure activity resulting in exaggerated cellular uptake of Ca++ (24). These mechanisms of cytotoxic edema related to epileptic activity are similar to those in AIS and could not explain the difference in the severity of cytotoxic edema in these two conditions. A possible explanation of this disparity, however, could be linked to the depolarization extent, which may be limited to a non-spreading depolarization in seizure activity as opposed to a terminal spreading depolarization in AIS (25). Cellular injury does not occur during the initial stage of a non-spreading depolarization. This might explain the reversibility of diffusion restriction in SE if it is self-limiting or treated timely and effectively. In AIS and in some patients with refractory or super-refractory SE, a terminal spreading depolarization occurs leading invariably to irreversible cellular injury.

### Limitations

In this study, we evaluated diffusion restriction in DWI sequence in patients with SE and AIS. We did not perform a regression analysis in order to assess the influence of clinical symptoms and EEG patterns on MRI features. We did not perform also an analysis related to other MRI sequences, such as perfusion imaging. To our view, these evaluations would be out of the scope of this study.

Another limitation of this study is a small cohort of patients, especially in the SE group. Therefore, reported cutoff values for DWI and ADC ratios warrant further prospective validations on greater cohorts of patients. However, to our best knowledge, this is the largest cohort of patients who underwent similar evaluation.

## Conclusion

Diffusion restriction was significantly more intense in patients with AIS as compared to those with SE. Quantitative analysis of DWI and ADC signals may be helpful in differentiating between AIS and SE when restricted diffusion is present on MRI.

## Data Availability Statement

The raw data supporting the conclusions of this article will be made available by the authors, without undue reservation.

## Ethics Statement

The studies involving human participants were reviewed and approved by the Ethics Committee of the Region of Salzburg, Austria (approval number 415-E/2422). The patients/participants provided their written informed consent to participate in this study. Written informed consent was obtained from the individual(s) for the publication of any potentially identifiable images or data included in this article.

## Author Contributions

LM, PB, TP, and GK contributed significantly to the conception and design of the presented paper, acquisition, analysis, interpretation of the data, and drafting of the paper. JS, GZ, AÖ, and SP contributed to the acquisition and analysis of data and revising of the paper for intellectual content. JP, ET, and MM contributed significantly to the conception of the study, interpretation of the results, and gave final approval of the submitted version of the manuscript. All authors contributed to the article and approved the submitted version.

## Funding

This study was supported by Fonds zur Förderung der wissenschaftlichen Forschung (FWF), Austrian Science Fund; Project Number KLI 969-B. GZ gratefully acknowledges the support of the WISS 2025 project IDA-Lab Salzburg (20204-WISS/225/197-2019 and 20102-F1901166-KZP).

## Conflict of Interest

The authors declare that the research was conducted in the absence of any commercial or financial relationships that could be construed as a potential conflict of interest.

## Publisher's Note

All claims expressed in this article are solely those of the authors and do not necessarily represent those of their affiliated organizations, or those of the publisher, the editors and the reviewers. Any product that may be evaluated in this article, or claim that may be made by its manufacturer, is not guaranteed or endorsed by the publisher.
